# White-Tailed Deer Response to Vehicle Approach: Evidence of Unclear and Present Danger

**DOI:** 10.1371/journal.pone.0109988

**Published:** 2014-10-15

**Authors:** Bradley F. Blackwell, Thomas W. Seamans, Travis L. DeVault

**Affiliations:** United States Department of Agriculture, Animal and Plant Health Inspection Service, Wildlife Services, National Wildlife Research Center, Ohio Field Station, Sandusky, Ohio, United States of America; University of Tasmania, Australia

## Abstract

The fundamental causes of animal-vehicle collisions are unclear, particularly at the level of animal detection of approaching vehicles and decision-making. Deer-vehicle collisions (DVCs) are especially costly in terms of animal mortality, property damage, and safety. Over one year, we exposed free-ranging white-tailed deer (*Odocoileus virginianus*) to vehicle approach under low ambient light conditions, from varying start distances, and vehicle speeds from 20 km/h to approximately 90 km/h. We modeled flight response by deer to an approaching vehicle and tested four hypotheses: 1) flight-initiation distance (FID) would correlate positively with start distance (indicating a spatial margin of safety); 2) deer would react to vehicle speed using a temporal margin of safety; 3) individuals reacting at greater FIDs would be more likely to cross the path of the vehicle; and 4) crossings would correlate positively with start distance, approach speed, and distance to concealing/refuge cover. We examined deer responses by quantiles. Median FID was 40% of start distance, irrespective of start distance or approach speed. Converting FID to time-to-collision (TTC), median TTC was 4.6 s, but uncorrelated with start distance or approach speed. The likelihood of deer crossing in front of the vehicle was not associated with greater FIDs or other explanatory variables. Because deer flight response to vehicle approach was highly variable, DVCs should be more likely with increasing vehicle speeds because of lower TTCs for a given distance. For road sections characterized by frequent DVCs, we recommend estimating TTC relative to vehicle speed and candidate line-of-sight distances adjusted downward by (1-P), where P represents our findings for the proportion of start distance by which >75% of deer had initiated flight. Where road design or conservation goals limit effectiveness of line-of-sight maintenance, we suggest incorporation of roadway obstacles that force drivers to slow vehicles, in addition to posting advisory speed limits.

## Introduction

Animal-vehicle collisions pose not only mortality to the animals involved, but in some cases substantial safety concerns to people and costs associated with collisions. Deer (Cervidae), given their size, general abundance, and association with humans, represent a particularly critical safety problem relative to collisions with vehicles. As an indirect example, based on its claims data State Farm Insurance Company estimates that 1.23 million deer-vehicle collisions (DVCs) occurred in the USA from July 1, 2011 through June 30, 2012, a 7.7% increase over the previous year and representing approximately $4 billion in damages annually (i.e., $3,305 U.S. per incident; https://www.statefarm.com/about-us/newsroom/2012/10/23/deer-vehicle-confrontations. Accessed 2014 Sep 19). Recent studies have elucidated habitat and landscape-level attributes that might increase the likelihood of DVCs and other animal-vehicle collisions (e.g., obstructive cover proximate to roads [1], [2], [3]; landscape diversity near roads, including lower densities of people [4], [5], [6]). Further, in areas of relatively high human density, but where human presence is essentially non-threatening, wildlife generally show shorter flight-initiation distances (FIDs) [7]. In the context of DVCs, shorter FIDs relative to vehicle approach equate to less time for the deer and driver to react. However, even with a wealth of information on near-road habitat factors that contribute to DVCs, effective reduction of these incidents is multifaceted and at present there is no economically and logistically feasible solution to the problem over large geographic scales.

Contributing to the difficulties in managing DVCs and other animal-vehicle collisions is a general lack of understanding about the fundamental causes of such collisions, particularly at the level of animal detection of approaching vehicles and subsequent decision-making [8]. Behavioral response of ungulates to road-based vehicles can vary relative to age, sex, antler presence/size [9], [10]; traffic volume/speed [10], [11], [12]; vehicle type and noise [13]; habitat features [3], [5], [9], [11], [12], see also [14]; and prior experience (hunting pressure) [15], [16]. We suggest, however, that commonalities in species-specific behavioral adaptations for predator detection and avoidance can provide insight into aspects of DVCs, *sensu* [17]. For example, white-tailed deer (*Odocoileus virginianus*) in dense vegetation might flee even when a predator is at considerable distance, likely because of loss of visual contact [18]. In contrast, alert and flight-initiation distances for this species are generally positively correlated with distance to an observed, potential threat, such as the point in which an observer begins an approach [18]. Thus, maintenance of visual contact with a potential threat is key to the white-tailed deer’s ability to make flight decisions based on relative speed and position of the threat.

Still, to effectively exploit deer antipredator behavior [19] relative to vehicle approach, deer must perceive vehicles as threats at some point during an approach. Indeed, recent evidence involving deer response to the approach of a ground-based vehicle [20], as well as bird response to aircraft [21], suggests that antipredator behavior theory is applicable to how deer respond to vehicles [17]. For example, Behrend & Lubeck [15] reported enhanced flight response by white-tailed deer to approach by road-based vehicles versus canoe, an indication that the approach of non-predator/human objects might stimulate differential levels of perceived risk or threat [7]. Horejsi [22] reported that flight response by barren ground caribous (*Rangifer tarandus groenlandicus*) to vehicle approach was positively correlated with distance of encounter and distance of closest approach.

There is also potential for the threat perceived by deer to be enhanced [7], [17], [23], [24]. For example, in a test of Helfman’s [25] *Threat Sensitivity Hypothesis*, FID by Columbian black-tailed deer (*O. hemionus columbianus*) increased relative to an increased pace of an approaching human and directness of the approach [26]. Time spent assessing the threat decreased with approach speed, but was positively associated with the distance at which the deer first became alert.

We recognize, however, that the perceived threat posed by a human versus a vehicle, as well as the subsequent responses, might differ depending upon experience, particularly if humans and their use of vehicles are associated with hunting [15], [27]. However, even when assuming experience with vehicles in context of traffic flow alone, animal responses are not necessarily predictable [8]. For example, in work with four species of macropods exposed to vehicle approach, Lee, Croft & Ramp [28] found that species with the lowest average FID had the lowest collision frequency, and those species with the highest average FID had the higher collision frequencies. Those species traveling shorter distances during flight fared better than those exhibiting longer travel distances. Notably, the probability of flight was higher at lower vehicle speeds, possibly due to enhanced, perceived threat resulting from a more “methodical approach” [29].

We questioned how vehicle speed and other ambient and herd-related factors might affect flight responses by free-ranging white-tailed deer and tested four hypotheses related to this question. First, we predicted that FID would correlate positively with start distance [29], [30], suggesting a spatial margin of safety [31]. Alternatively, there is evidence that predator approach speed exerts a positive effect on FID in some species [16], [27], indicating a temporal margin of safety. We also predicted that individuals reacting at greater FIDs would be more likely to cross the path of the vehicle [28], which we consider as an index of collision likelihood. Finally, we predicted that crossing frequency would be positively correlated with approach speed, start distance, and distance to concealing cover [32]. Our objective was to model flight response by free-ranging deer at night relative to speed of an approaching vehicle, environmental conditions, and seasonal ecology of deer.

## Materials and Methods

### Study Area

We conducted our experiment at the National Aeronautics and Space Administration (NASA) Plum Brook Station (PBS), Erie County, Ohio, USA (41^o^ 22′ N, 82^o^ 41′ W). The 2200-ha PBS is enclosed by a 2.4-m high chain-link fence with barbed-wire outriggers. Habitat within PBS differs from the surrounding mix of agricultural and suburban area, comprising canopy-dogwood (*Cornus* spp.; 39%), old field and grasslands (31%), open woodlands (15%), and mixed hardwood forests (11%) interspersed by abandoned and actively used structures relating to NASA and prior operations, and paved roads that circle and bisect the station. Deer ingress and egress occurs through several gaps between the fence and ground. Further, deer on PBS are routinely exposed to vehicles during daylight hours (at a maximum speed of 65 km/h) and, to a lesser degree, at night. In addition, roads on PBS are generally bordered by a mown strip approximately 30 m in width, reducing the potential for DVCs due to visual obstruction near the roadside. However, during our study, at least two DVCs occurred (TWS, pers. obs.). Estimated deer density during winter 2012 through 2013 was 15 individuals/km^2^ (0.15 individuals/ha; U.S. Department of Agriculture, Wildlife Services, Ohio program, unpublished data). Relative to animal condition, a factor that can affect sensitivity to threat [33], we observed no deer that appeared malnourished. Further, the PBS herd has access to natural vegetation resources on site as well as suburban vegetation and agricultural crops on bordering properties. Also, because the PBS herd is not a closed population, these deer are exposed to roads with greater traffic volume and area than on PBS. Thus, we consider these animals as representative of suburban white-tailed deer herds throughout the contiguous USA.

In addition, the PBS herd has been exposed to controlled hunts (males and females) during fall and winter for 15 years. Selected hunters on foot are assigned to specific sectors across PBS. Depending upon number of hunters relative to sectors, limited hunting is also conducted by locating deer via vehicle. In such cases, hunters are typically offloaded, but there are instances in which hunters use the stationary vehicle as a shooting platform.

### Experimental Design

We conducted our experiment from 4 April 2012 through 15 April 2013 and our observations were interrupted by two days of the 2012/13 NASA controlled hunt. During our study, the PBS herd was hunted on 8 December and we conducted observations on 12 December, recording three approaches to deer. The next hunt occurred on 05 January 2013, but we did not resume the experiment and record subsequent observations until 15 January 2013. Because of possible biases associated with the hunts, we examined our data relative to pre- and post-hunt vehicle approaches prior to formal analyses (see below).

We selected four routes on PBS (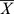 = 4.9 km; range = 4.7−11.6 km), that allowed us distance to accelerate to a maximum speed between 20 and 90 km/h during approaches toward free-ranging deer. Our adherence to a pre-planned route allowed us to reduce the likelihood of double sampling on the same date by noting direction of travel for animals responding to our approach, and avoiding subsequent route sections within 0.5 km of the last approach. We were restricted to a maximum speed of 90 km/h for safety concerns. All vehicle approaches were made with headlamps on high beam. Our approach vehicle was a Ford F250 pickup truck fitted with a Forward Looking Infrared Camera (PATHFINDIR, thermal imaging system, FLIR Systems, Inc., Goleta, CA, USA) mounted atop the cab. The camera was mounted inside a rotate/tilt housing that was controlled remotely from within the truck and in reference to a video feed from the camera. We could easily detect deer at night up to ∼800 m.

Initially, we planned to complete one randomly selected morning (i.e., prior to sunrise) of data collection per week over one year. However, after two approaches over two days we realized that early-morning traffic arriving to PBS presented additional safety concerns with respect to high-speed approaches. Subsequently, we shifted our observations to begin 30 min after sunset. We retained the data from two morning approaches in our data set, as both were conducted under dark conditions and prior to traffic increases on PBS. Also, we maintained a protocol of data collection of at most once per week, but weather events and the PBS hunt extended the mean interval between observation nights.

We randomized order of travel relative to route, direction, and maximum approach speed. A driver and observer were present during data collection. We restricted our observations to a single approach on a route where our approach was potentially visible to deer ≤1 km away on the same road or sections of a proximate route. In addition, we restricted observations to clear conditions and when the road surface was dry, thus preventing additional noise on approach due to a wet road surface or the effects of snow and ice. We attempted to limit our data collection to periods when wind speeds were ≤6 km/h, but higher gusts occurred during some approaches.

We drove each route at approximately 20 km/h until a deer or group was sighted on the FLIR. When a single deer or at least one member of a clearly contiguous group was detected via camera on or between the road edge and power lines bisecting the grass median (within approximately 5 m of the road), the observer noted the location of the deer relative to the road and dropped a marker from the truck window denoting the start point. More specifically, we attempted to restrict our observations to animals on or within 5 m of the road edge, but some variation was inevitable given ambient lighting and viewing through a camera. The driver then began the approach by quickly accelerating from our original speed (e.g., ≤20 km/h) to the pre-selected maximum approach speed, attempting an approach at a randomly-selected maximum speed of either 40 km/h or 90 km/h. However, maximum approach speed (hereafter, approach speed) was inherently affected by initial distance between the truck and animals. Further, on occasion the image from the camera was not clear and we came to a stop before concluding that a deer or group was present. As approach speed and change in approach speed are also critical components in how a prey species interprets the intensity of threat posed by a predator [7], [23], [34], and the potential behavioral effect from the initial acceleration might differ with distance [26], we recorded the start speed and start distance for each approach (see below). We did not initiate an approach when the individual or group was bedded.

We were not able to quantify accurately and consistently aspects of alert behavior by an individual or group in response to the approach of the truck, under low-ambient light conditions, at distance, and with movement of the vehicle. Therefore, we focused on flight behavior. Specifically, the observer dropped a marker from the truck window at the instant an individual deer initiated movement to avoid the truck. We considered flight response as a behavior that would eventually take an individual deer away from the road and contact with the road edge (i.e., avoiding collision), or flight away from its initial off-road position, before the truck was perpendicular with the position of the individual or central point of the group at initial sighting. When approaching a group we recorded data for as many individuals in the group as possible. We considered the approach complete when the truck was perpendicular to the original position of the individual or group. We did not decelerate until after passing the original position of the individual or group, but we continued observation of the deer to determine if it crossed the road after we passed.

Subsequent to each approach, we recorded the distance between the marker denoting the moment at reaction and the original position of the deer or original central point of the group (i.e., ≥2 animals), defined as the FID, and the distance between the original position of the deer or group and the approach start point on the route (i.e., the start distance). We used a Bushnell Yardage Pro 1000 Laser Ranging System (Overland Park, Kansas, USA) to measure distances to the nearest m. Because the vehicle was illuminated by the headlamps on high beam, and sound from the truck inevitably increased with decreasing distance to deer, we considered start distance as a detection distance, but also recognized the possibility that some approaches might fall outside a deer’s zone of (threat) awareness [26]; see also [30].

We adjusted our start distance and FID measurements by a correction factor for the forward speed of the marker when dropped and based on our recorded speeds at start points of approaches and approach speeds [20], [35]. Because of limitations in accurately measuring start distances >1 km, we decided beforehand to record start distance as 1001 m for such approaches [20]. Similar to Lee et al. [28], we recorded whether the deer or group crossed the road in front of the vehicle. If an individual or group failed to react, the FID and frequency of crossing were recorded as 0 m and zero, respectively. We recorded group size as the number of animals within the contiguous group present during the approach, not simply those animals reacting. We note that group size in this context does not infer overall size of the foraging group, as animals away from the road and possibly associated with the focal animal/group likely went unobserved. As an extension of FID, we also calculated an additional response variable, time-to-collision (TTC) from point of flight, as
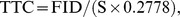
where S represents the approach speed (km/h) and 0.2778 the adjustment to m/s [35].

In addition to distance data, we recorded ambient light intensity (µmol m^−2 ^s^−1^) with a Li-Cor LI-250 Light Meter and LI-190SA Quantum Sensor (Lincoln, Nebraska, USA), and wind speed and air temperature with a Kestrel 4500 Pocket Weather Tracker (Nielson-Kellerman, Boothwyn, Pennsylvania, USA). We recognize also that gender of the individual(s) can affect FID, but accurate and consistent identification of gender was not possible, given the constraints on our observations (noted above). Instead, we examined the distribution of FIDs and group size via a seasonal component by which gender effects (particularly associated with breeding) on FID might be expressed. We defined season as post-rut (December–April), calving/pre-rut (May–August), and rut (September through November). As for age effects, individuals recognized clearly as fawns were noted, as were females with fawns, but neither observation type was included among our data for analyses. Vigilance of fawns differs from that of adults [36] and response of females with fawns was expected to differ from that of females absent dependent fawns [16].

Adequate replication of habitat effects, within the context of PBS, was impossible given that we had no foreknowledge of where we might encounter deer. We therefore included an index of distance to concealing or refuge cover (cover distance) from the initial position of the animal or group. Here, cover distance represented shrub vegetation or timber that could conceal a standing deer. Vigilance in ungulate species generally increases with decreasing distance to visual obstruction (i.e., cover characteristics that might conceal a predator [37], [38], but FID also tends to increase with increasing distance to concealing or refuge cover [7].

### Analyses

We considered each vehicle approach as an experimental unit. We removed from our analyses three approaches, begun from 568 m to 963 m, because of operational inconsistencies during the approaches and FIDs that differed little from the remaining data. We considered these data indicative that our start points were likely beyond the animals’ perception of threat [26], [30]. We omitted another approach because of driver error during the approach. Our sampling protocol resulted in >84% of data collection occurring prior to the hunts on PBS, and we found no evidence that FID differed between pre- and post-hunt (see Figure S1 and below).

We examined seven factors pertaining either to ambient conditions or herd-related metrics that might potentially affect deer response to vehicle approach and, thus, serve as covariates in our modeling of FID and TTC. Because our approach speed varied relative to start distance, a comparison of these seven factors by speed was not possible. However, our median start distance was 193 m (range = 62–438 m), therefore we examined these factors relative to an arbitrary start distance category (“short” approaches: vehicle start distance ≤200 m from the individual or group; “long” approaches: >200 m). These data were not normally distributed; therefore we used the Wilcoxon Two-Sample Test for the comparisons. Also, because of potential effects of ecological season on deer response to vehicle approach, we compared approach speeds and FIDs by the three pre-defined seasonal periods, noted above. These data were also not normally distributed; here, we used the Kruskal-Wallis Test for both comparisons. Given that reactions among animals within a group were likely not independent [39] and not all individuals within a group reacted, we calculated the median FID per group (as opposed to the arithmetic mean) and, by extension TTC, as response variables for our analyses [35].

In addition, because acceleration could have differentially affected responses relative to approach speed, we sorted our data by short and long start-distance categories (see above). Importantly, an animal might have reacted sooner to the vehicle accelerating to higher speeds over a start distance ≤200 m, but this reaction might not be evident in the examination of absolute FID relative to FIDs in response to approaches from >200 m. Therefore, we calculated the proportion of start distance represented by the FID per approach. We used the Wilcoxon Two-Sample Test to compare this proportion for short approaches to those begun at >200 m, but for approaches ≥60 km/h only. Limiting our comparison to approaches conducted at ≥60 km/h allowed us to better balance our sample sizes between start-distance categories. Further, by controlling for approach speed, if acceleration effects were confounding the potential effect of start distance or other independent variables on FID, we expected that deer exposed to short approaches would have reacted sooner (i.e., at a greater proportion of start distance) than those exposed to approaches from >200 m. Also, because our start distance varied among approach speeds, we report the proportion of start distance represented by FID (i.e., FID/start distance) and relative to start distance.

Based on our examination of ambient conditions and herd-related factors (Table S1), we modeled the effects of start distance, approach speed, and start distance x approach speed on FID and TTC. We considered that the effect of start distance x approach speed with the individual effects of start distance and vehicle speed, respectively, could provide further indication as to possible confounding effects of acceleration during the approach (e.g., a significant negative correlation of the interaction, but no effect of start distance; see also interacting effects on flight [40], [41]. Also, our population comprised animals of various ages, thus unmeasured levels of exposure to vehicles or predation events, and possibly differing in individual tendencies with respect to flight behavior [42] and boldness [43], could potentially have affected FID and TTC.

We used quantile regression (PROC QUANTREG; SAS 9.2, SAS, Inc., Cary, NC, USA) to model FID, TTC, and frequency of crossing. Quantile regression is an extension of the linear model and is used for estimating rates of change (slopes) in all or specified parts of the distribution of the response variable [44], [45]. We note that the more traditional measures of central tendency in response variables (e.g., via generalized linear models) might not adequately reflect individual tendencies relative to perceived threat. Further, the nature of our experimental design likely violated assumptions associated with models based on central tendency of response variables. For example, by definition start distance >FID, therefore the potential exists for a non-biological, statistically positive relationship between the two metrics, such that there is increasing variance as values of the variables increase [46]. If so, any assumption of homoscedasticity is violated. Also, deer responses to vehicle approach might have been affected not simply by sound associated with acceleration, but the change in speed over the approach distance to the point of maximum acceleration. Again, an assumption of homogeneity of variance associated with the distribution of FID or TTC relative to independent variables is questionable. Other unmeasured factors (e.g., presence of predators or recent predation events) might have enhanced perception of threat [8], or produced immeasurable and complex interactions that yielded unequal variation [44], [45], (see also Appendix S1).

Also, we examined the correlation between the proportion of start distance represented by FID (i.e., FID/start distance) and frequency of crossing via the Pearson Product Moment Correlation. We restricted this analysis only to instances where the median FID>0 and we standardized the number of deer crossing by the group size for each observation. Further, we modeled the effect of start distance, approach speed, and cover distance on the frequency of crossing (standardized by group size) using quantile regression.

We evaluated results of all analyses at α = 0.05. The lack of replication across multiple herds dictates that our inference is relative to PBS.

### Ethics Statement

The authors secured permission from NASA to conduct the research on PBS via their review of National Wildlife Research Center QA-1922 and personal communication (PBS phone: +1 419.621.3236). The Institutional Animal Care and Use Committee of the United States Department of Agriculture, Animal and Plant Health Inspection Service, Wildlife Services, National Wildlife Research Center approved all procedures used in this study (QA-1922). No animals were injured or killed during the conduct of this study.

## Results

We completed 67 approaches over 36 nights during the year of the experiment, with an average of 2 approaches per night (SD = 1.2 approaches), and a mean interval between experimental sessions of 11 nights (SD = 7.8 nights). With the omission of the four approaches noted above, we included 63 approaches in our analyses. Relative to pre- and post-hunt data, we found no evidence of overt sensitivity to our vehicle for post-hunt approaches (pre-hunt approaches: n = 53, median FID/start = 0.42, range = 0.0–0.98; post-hunt approaches: n = 10, median FID/start = 0.35, range = 0.0–0.99; Figure S1).

Because we encountered deer by chance and on some route sections acceleration to approximately 90 km/h was difficult, our distribution of approach speeds was greater at middle speeds (40 km/h: n = 18 approaches; >40 km/h but <80 km/h: n = 28 approaches; 80−89 km/h: n = 17 approaches); 2 approaches were conducted at 20 and 25 km/h, respectively (see also Appendix S1). Despite weather conditions that prevented us from conducting approaches, particularly rain and snow events during November through February, our allocation of approach speeds was similar among seasonal periods (rut: n = 16 approaches, median approach speed = 56 km/h, Sum of scores = 451.0, Expected = 512.0; post-rut: n = 12 approaches, median = 56 km/h, Sum of scores = 350.0, Expected = 384.0; calving/pre-rut: n = 35 approaches, median = 62 km/h, Sum of scores = 1215.0, Expected = 1120.0; df = 2, *P* = 0.4046). Further, we observed no difference in FID by seasonal period (rut: median FID = 70.5 m, Sum of scores = 386.5, Expected values noted above; calving/pre-rut: median = 94.7 m, Sum of scores = 1273.0; post-rut: median = 79.4 m, Sum of scores = 356.5; *P* = 0.0770).

Only ambient light differed by start-distance category, with a greater intensity measured for approaches beginning at >200 m (Table S1). However, given the mode light intensity was 0 µMol m^−2 ^s^−1^ for both distance categories, we consider this difference as not biologically significant with respect to deer discerning an approaching vehicle with headlamps on high beam. By comparison, partly cloudy to full-sun conditions in the same experimental area have previously been measured at ∼550 to >1800 µMol m^−2 ^s^−1^ [47].

**Figure 1:**
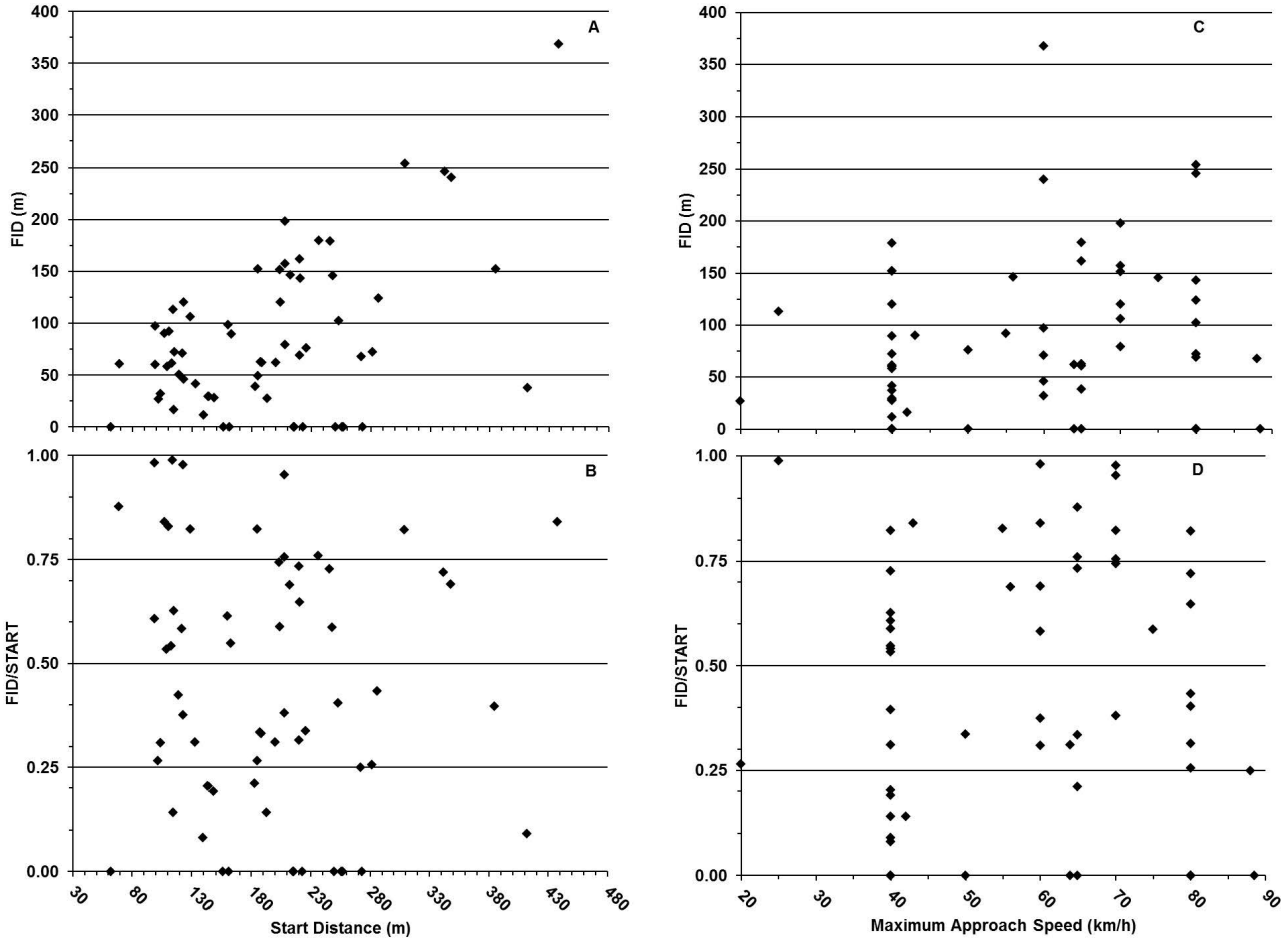
Responses by white-tailed deer to vehicle approach from varying start distances during an experiment conducted in Erie County, Ohio, USA (41^o^ 22′ N, 82^o^ 41′ W), from 14 April 2012 through 15 April 2013: (A) Flight-initiation distance (FID) relative to start distance; (B) Ratio, FID/start distance, relative to start distance; (C) FID relative to appoach speed; and (D) FID/start distance relative to approach speed. See text for definitions of FID and start distance relative to this experiment.

We found that 76.2% of deer/groups (n = 48) exhibited an FID from 1−180 m, 7.9% of FIDs (n = 5) were >180 m, and 17.5% of FIDs (n = 11) were <12 m (10 of which occurred at 0 m; overall range = 0.0–368.1 m, median = 69.1 m), but there was no trend (Figure 1A). Because our start distance varied among approach speeds, we also examined the proportion of start distance represented by FID (i.e., FID/start distance) and relative to start distance, and found 29 approaches (46.0%) in which the FID/start was ≥0.50 (median FID/start distance = 0.40, range = 0.00–1.00, N = 63 approaches; Figure 1B). Similar to the relationship of FID to start distance, there was no trend in FID relative to approach speed (Figure 1C). Further, for the 28 approaches conducted between 40 and 80 km/h we found that the median FID/start distance = 0.65. By contrast, approaches from 20 to 40 km/h and approaches ≥80 km/h yielded measures of the median FID/start distance = 0.35 and 0.31 respectively, indicating little to no effect of approach speed on deer FID (Figure 1D).

In addition, our median start distance was approximately 50 m greater for fast approaches as a result of the distance required to reach higher speeds (approach speeds <60 km/h: min start distance = 99.3 m, median = 154.6 m, maximum = 412.0 m; approach speeds ≥60 km/h: min = 62.0 m, median = 208.0 m, maximum = 428.0 m). However, we observed no statistical difference in FID/start distance between long and short approaches conducted at 60–89 km/h (long approaches: median FID/start distance = 0.49, Sum of scores = 409, Expected = 399, n = 21 approaches; short approaches: median FID/start distance = 0.46, Sum of scores = 294, Expected = 304, n = 16 approaches; df = 1, *P* = 0.7721; see also Figure 1B). Thus, we consider that acceleration over approaches ≤200 m did not confound our interpretation of FID relative to other parameters in our model (see below).

**Figure 2:**
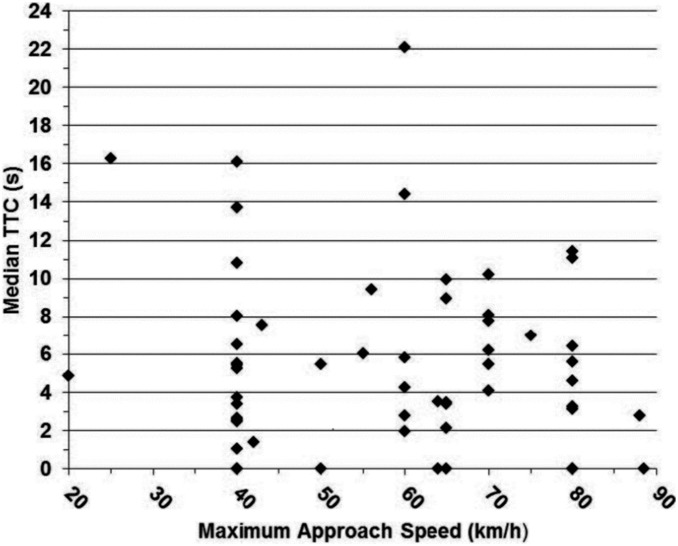
Time-to-collision (TTC) for white-tailed deer responding to vehicle approach relative to vehicle approach speed during an experiment conducted in Erie County, Ohio, USA (41^o^ 22′ N, 82^o^ 41′ W), from 14 April 2012 through 15 April 2013. See text for experimental protocol.

With regard to our models for FID and TTC, we found no effects of approach speed, start distance, or start distance x approach speed on either response variable (Table 1; Figures 2, S2, & S3).

**Table 1:** Parameter estimates relative to multivariate-model effects on FID^1^ and TTC^1^ for white-tailed deer responding to vehicle approach at varying speeds (20 km/h to approximately 90 km/h) during an experiment conducted in Erie County, Ohio, USA (41^o^ 22′ N, 82^o^ 41′ W), from 14 April 2012 through 15 April 2013.

Response Variable	Parameter^1^	Estimate	SE	t	P
FID	Intercept	65.2247	94.5687	0.69	0.4931
	Start distance	−0.2566	0.4942	−0.52	0.6056
	Approach speed	−1.0086	1.6984	−0.59	0.5549
	Start distance×Approach speed	0.0040	0.0086	0.46	0.6467
TTC	Intercept	5.9146	8.8920	0.67	0.5085
	Start distance	−0.0233	0.0484	−0.48	0.6326
	Approach speed	−0.0915	0.1443	−0.63	0.5285
	Start distance×Approach speed	0.0004	0.0002	0.49	0.6270
Crossingfrequency^1^	Intercept	0.5081	2.4354	0.21	0.8370
	Start distance	−0.0005	0.0082	−0.06	0.9506
	Approach speed	0.0002	0.0256	0.01	0.9933
	Cover distance	0.0026	0.0586	0.04	0.9651

Summary statistics are based on quantile regression via Interior Point algorithm. Because no parameters exerted statistically significant effects, only findings for the top 90% of responses (i.e., 0.10 quantile) are shown (see also Figures S2 & S3).

1 See text for definitions.

We also observed 23 instances (36.5% of approaches) of deer crossing the path of the vehicle, 8 (33%) of which involved animals positioned between the road and a fence line (i.e., concealing/refuge cover was across the road). There was no correlation between frequency of crossing (i.e., number crossing/group size) and FID/start distance (N = 53 approaches where FID>0, r = −0.1153 *P* = 0.6003). Also, we found no statistical effect of start distance, approach speed, or cover distance on frequency of crossing (Table 1).

## Discussion

We predicted that FID by free-ranging white-tailed deer in response to vehicle approach would correlate positively with start distance (indicating a spatial margin of safety), but also assessed the alternative hypothesis of a positive correlation with approach speed or temporal margin of safety. However, 50% of deer initiated flight within a median distance equal to 40% of vehicle start distance and with no effect of approach speed, and no effect of start distance or its interaction with approach speed. We found similar results for TTC. We suggest that vehicle approach under typical roadway conditions (e.g., absent hunting from the vehicle or use of a vehicle in pursuit of an animal) likely does not enhance threat perceived by deer until animal-to-vehicle distance is <470 m. After this point, which might represent the zone of awareness [26] for white-tailed deer, our findings indicate that FID is not dynamically adjusted with start distance and is highly variable within the population [31]. We recognize, also, that individual, behavioral tendencies relative to threat [42], [43], (Appendix S1), as well as other unmeasured factors influencing perceived threat [8], might also be represented in our data, as indicated by the substantial variation present around the median flight responses (Figure 1A). In essence, however, neither longer approach distances nor higher approach speeds elicited earlier flight responses by deer in our study.

Our findings contrast to those by Lee et al. [28] for macropods, in that we observed no correlation between proportionately greater FIDs relative to start distance and frequency of crossing. Moreover, neither vehicle approach speed nor greater start distances resulted in statistically significant flightiness or protean behavior [48], [49]. We recognize, also, that distance to concealing or refuge cover figures prominently as a factor contributing to flight in other taxa [7] and the relative consistency of this parameter (cover distance) in our experiment (Table S1) might have contributed to our findings with regard to spatially-based flight decisions.

We must question, however, whether deer or other animals can adequately process visual stimuli associated with vehicle approach, particularly as related to approach speed. For example, Whittington, St. Clair & Mercer [50] suggested that to wolves (*Canis lupus*), vehicles likely appear relatively static as compared to the body motions associated with animal and human movement. Consequently, it might be difficult for wolves to gauge the speed of vehicles (or threat), particularly on large, smooth highways. The implication is that misinterpretation of vehicle speed, and subsequently the distance of the threat, is a factor in wolf-vehicle collisions. Further, recent findings by DeVault et al. [35] lend support to the role of misinterpretation of vehicle speed in animal-vehicle collisions, but to a degree. Specifically, DeVault et al. [35] found a differential response by turkey vultures (*Cathartes aura*) to vehicle approach speed between 30 km/h and 90 km/h, one that evidenced a possible ill adaption to 90-km/h approaches, the highest vehicle speed tested.

A poor response to fast-approaching vehicles might be a manifestation of how visually-oriented animals process light stimuli relative to detecting and responding to approaching objects. Specifically, DeVault et al. [35], and citations therein, noted that sensory and neural mechanisms dedicated to processing visual stimuli, particularly the near exponential growth of the angle subtended by the approaching vehicle on the retina of the animal (i.e., the looming effect), are adapted to detect predators, other animals, and natural objects, and thus likely inadequate for detection and response to fast-approaching vehicles.

Further, unlike our experiment, the DeVault et al. [35] experiment was conducted during daylight hours, conditions which would enhance detection and possibly affect flight decisions [28]. Also, differential responses by white-tailed deer to vehicle approach at night relative to vehicle lighting treatments [20] are indicative that vehicle lighting serves as the main stimulus for the approaching “object”, as opposed to the vehicle proper (Appendix S2). Further, though it is unlikely that a dark-adapted deer visual system is overwhelmed by vehicle lighting [20], the looming effect of the smaller-area light source is likely less than that of the larger vehicle [35]. Considering the reasoning by Whittington et al. [50] and DeVault et al. [35] relative to animal processing of visual stimuli associated with speed of vehicle approach, we agree that at some point vehicle approach speed will overcome an animal’s ability to detect and react effectively to an impending collision. However, what is important to the development of management to reduce DVCs is an understanding of how flight decisions are made (e.g., spatially or temporally) within a particular road context, what factors contribute most to the decision process, and how those factors can be most effectively exploited.

### Management Considerations

The frequency at which individual deer encounter vehicle traffic without injury or undue stress will inherently affect potential habituation to vehicle approach (e.g., degree of exposure to non-threatening human disturbance [16]). However, when detected and on a direct approach, vehicles can elicit antipredator behavior in white-tailed deer [20] and other taxa [17], [21], [22]. Considering the potential safety issues with regard to animal size and collision energy, how deer respond to vehicle approach has implications for planning with regard to vehicle speed in areas experiencing frequent DVCs.

Specifically, if deer reaction to vehicle approach is not associated with vehicle speed, time-to-collision must, with increasing speed, decrease for a given approach distance. Logically, kinetic energy imparted to animal and vehicle resulting from DVCs at greater speeds will also increase. Further, vehicle speed and poor visibility due to obstruction or ambient light serve to decrease driver reaction to an impending DVC [51]. We recommend, therefore, that municipalities, parks, and wilderness areas that have roads characterized by frequent DVCs work proactively to reduce vehicle speeds. As per Huijser et al. [52], there are three main ways for authorities to reduce vehicle speeds within high animal-vehicle collision areas: (1) reduce the posted speed, (2) reduce the design speed (i.e., the speed based on the geometric features of the roadway) through traffic management or redesign, and (3) post an advisory speed (i.e., a speed lower than the posted speed for the roadway and based on site-specific conditions). Options 1 and 3 are realistic only if posted speed limits are enforced. If option 2 is reasonable from the perspective of habitat manipulation, we suggest that planners consider when deer will first detect the oncoming vehicle (i.e., the start or detection distance relative to a road section characterized by frequent DVCs). Using our findings as a working example, one can estimate TTC based on a managed line-of-sight of distance D (m) and a select posted or advisory speed S (km/h) as
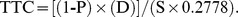



The contribution of vehicle speed (adjusted to m/s via the 0.2778 multiplier) to TTC is mathematical only, as we observed no effect of speed on FID. The parameter P is based on our findings for FID/start distance. For median FID/start distance = 0.20 we found that >75% of deer had initiated flight at or before the vehicle had traveled 80% of the start distance. Therefore, management of D at 90 m, incorporating P = 0.2, and assuming speed at 90 km/h, yields TTC = 2.9 s.

We recognize that option 2 could entail substantial financial input and might pose problems in wilderness areas or parks relative to increased habitat destruction and disturbance. In these instances, the design speed could be lowered possibly at less expense via incorporation of obstacles (e.g., speed bumps) that force drivers to slow their vehicles. Constructed obstacles would also aid in situations where the roadway curves, thus reducing visibility for deer and drivers. Importantly, we suggest that these actions be integrated into an overall planning and management effort designed to stem animal-vehicle collisions [6], [50], [53], [54]. Finally, opportunities to revisit the research approach described herein to incorporate daylight and nighttime approaches, variation in distance to refuge or concealing cover, as well as speeds >90 km/h, would provide more specificity in management recommendations (Appendix S1), particularly for parks and wilderness areas where management of roadside habitat might be less desirable.

## Supporting Information

Figure S1 **Proportion of start distance represented by FID (i.e., FID/start distance) for approaches conducted prior to controlled hunts on PBS (i.e., dashed vertical line represents the first hunt for the 2102/2013 season which took place on 8 December 2012) and afterwards.** See text for definitions.
(DOCX)

Figure S2 **Slopes and 95% confidence intervals by quantile for the effects of approach speed (SPD), start distance (START), and approach speed x start distance on the median FID (MEDFID) observed per group during an experiment conducted in Erie County, Ohio, USA (41^o^ 22′ N, 82^o^ 41′ W), from 14 April 2012 through 15 April 2013, in which free-ranging white-tailed deer were exposed to vehicle approach.** We selected the resampling option (which incorporates a Markov chain marginal bootstrap), and the Process option to obtain estimates of quantiles for each parameter. See text for definitions.
(DOCX)

Figure S3 **Slopes and 95% confidence intervals by quantile for the effects of approach speed (SPD), start distance (START), and approach speed x start distance on the median TTC (MEDTTC) observed per group during an experiment conducted in Erie County, Ohio, USA (41^o^ 22′ N, 82^o^ 41′ W), from 14 April 2012 through 15 April 2013, in which free-ranging white-tailed deer were exposed to vehicle approach.** We selected the resampling option (which incorporates a Markov chain marginal bootstrap), and the Process option to obtain estimates of quantiles for each parameter. See text for definitions.
(DOCX)

Table S1 **Parameters potentially affecting white-tailed deer response to vehicle approach under two approach-speed categories during an experiment conducted in Erie County, Ohio, USA (41^o^ 22′ N, 82^o^ 41′ W), from 14 April 2012 through 15 April 2013.**
(DOCX)

Appendix S1 **Assumptions & Constraints.**
(DOCX)

Appendix S2 **Deer Response to Vehicle Approach & Lighting.**
(DOCX)
