# Supplementary figures and images for: White-Tailed Deer Response to Vehicle Approach: Evidence of Unclear and Present Danger
Source: PLoS One. 2014 Oct 15;9(10):e109988. doi: 10.1371/journal.pone.0109988 (PMC4198184; doi:10.1371/journal.pone.0109988)

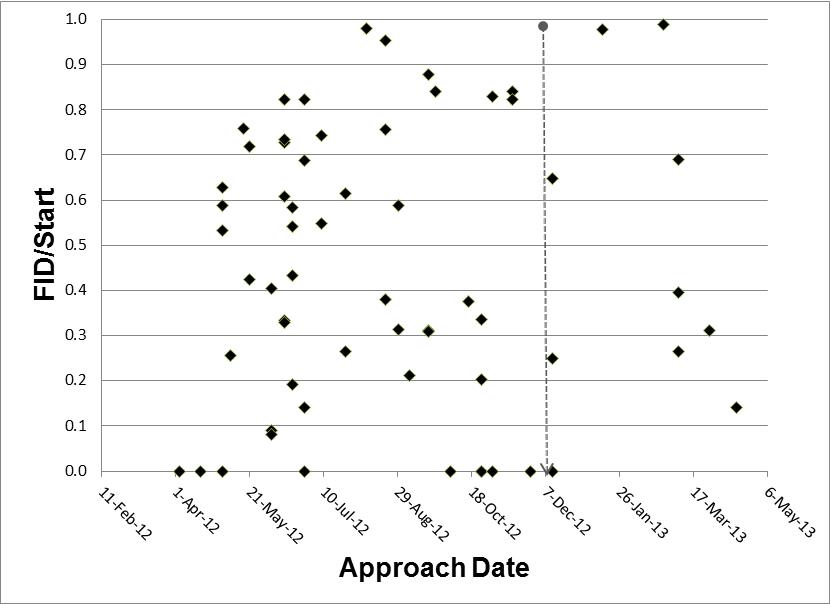

Supplement: Figure S1 — Proportion of start distance represented by FID (i.e., FID/start distance) for approaches conducted prior to controlled hunts on PBS (i.e., dashed vertical line represents the first hunt for the 2102/2013 season which took place on 8 December 2012) and afterwards. See text for definitions. (DOCX) [file pone.0109988.s001.docx]

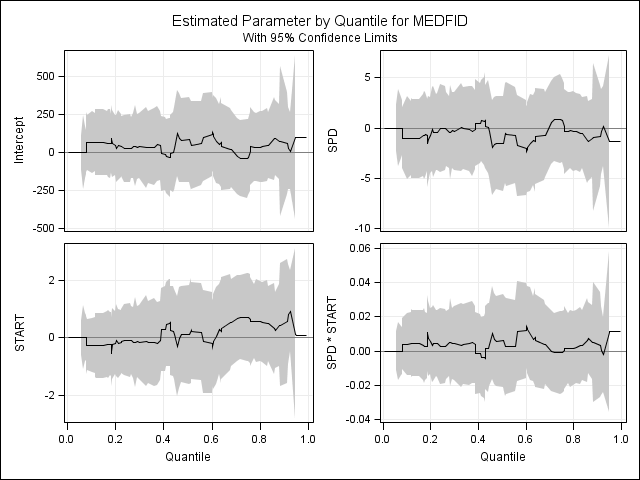

Supplement: Figure S2 — Slopes and 95% confidence intervals by quantile for the effects of approach speed (SPD), start distance (START), and approach speed x start distance on the median FID (MEDFID) observed per group during an experiment conducted in Erie County, Ohio, USA (41o 22′ N, 82o 41′ W), from 14 April 2012 through 15 April 2013, in which free-ranging white-tailed deer were exposed to vehicle approach. We selected the resampling option (which incorporates a Markov chain marginal bootstrap), and the Process option to obtain estimates of quantiles for each parameter. See text for definitions. (DOCX) [file pone.0109988.s002.docx]

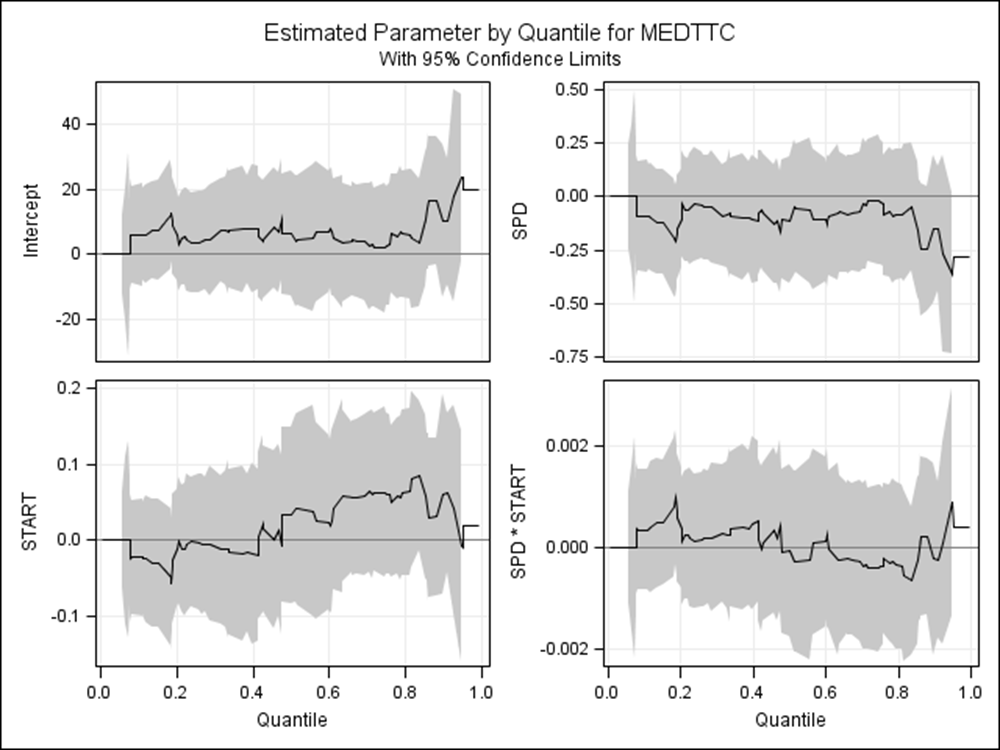

Supplement: Figure S3 — Slopes and 95% confidence intervals by quantile for the effects of approach speed (SPD), start distance (START), and approach speed x start distance on the median TTC (MEDTTC) observed per group during an experiment conducted in Erie County, Ohio, USA (41o 22′ N, 82o 41′ W), from 14 April 2012 through 15 April 2013, in which free-ranging white-tailed deer were exposed to vehicle approach. We selected the resampling option (which incorporates a Markov chain marginal bootstrap), and the Process option to obtain estimates of quantiles for each parameter. See text for definitions. (DOCX) [file pone.0109988.s003.docx]
