# Supplementary material for: White-Tailed Deer Response to Vehicle Approach: Evidence of Unclear and Present Danger
Source: PLoS One. 2014 Oct 15;9(10):e109988. doi: 10.1371/journal.pone.0109988 (PMC4198184; doi:10.1371/journal.pone.0109988)
Supplement: Table S1 — Parameters potentially affecting white-tailed deer response to vehicle approach under two approach-speed categories during an experiment conducted in Erie County, Ohio, USA (41o 22′ N, 82o 41′ W), from 14 April 2012 through 15 April 2013. (DOCX) [file pone.0109988.s004.docx]

|  |  |  |  |  |  | Wilcoxon 2-Sample Test^2^ | | | |
| --- | --- | --- | --- | --- | --- | --- | --- | --- | --- |
| Start Distance | Variable^1^ | N | Med | Min | Max | Sum of Scores | Expected | P |  |
| ≤200 m | Ambient light intensity (μMol m^-2^ s^-1^) | 33 | 0.0 | 0.0 | 7.2 | 895.0 | 1056.0 | 0.019 |  |
|  | Cover distance (m) | 33 | 18.0 | 0.0 | 101.0 | 1080.0 | 1056.0 | 0.747 |  |
|  | Group size | 33 | 1 | 1 | 5 | 980.50 | 1056.0 | 0.244 |  |
|  | Individual/herd distance from road (m) | 33 | 5.0 | 0.0 | 14.0 | 1025.0 | 1056.0 | 0.674 |  |
|  | Speed at start (km/h) | 33 | 20.0 | 0.0 | 40.0 | 1144.0 | 1056.0 | 0.216 |  |
|  | Temperature (C^◦^) | 33 | 18.9 | -2.2 | 29.4 | 1056.0 | 1056.0 | 1.000 |  |
|  | Wind speed (km/h) | 33 | 0.0 | 0.0 | 14.5 | 1139.5 | 1056.0 | 0.190 |  |
| >200 m | Ambient light | 30 | 0.3 | 0.0 | 8.3 | 1121.0 | 960.0 | * |  |
|  | Cover distance (m) | 30 | 17.8 | 2.0 | 50.0 | 936.0 | 960.0 | * |  |
|  | Group size | 30 | 1 | 1 | 8 | 1035.5 | 960.0 | * |  |
|  | Individual/herd distance from road (m) | 30 | 5.0 | 0.0 | 27.0 | 991.0 | 960.0 | * |  |
|  | Speed at start (km/h) | 30 | 20.0 | 0.0 | 40.0 | 872.0 | 960.0 | * |  |
|  | Temperature | 30 | 17.6 | 1.2 | 29.4 | 960.0 | 960.0 | * |  |
|  | Wind | 30 | 0.0 | 0.0 | 6.4 | 876.5 | 960.0 | * |  |
| ^1^See text for definitions.  ^2^Comparison of parameters between approach-speed categories. Expected sum of scores = 952.50 | | | | | | | | |  |
|  | | | | | | | |  |  |
