# Supplementary material for: White-Tailed Deer Response to Vehicle Approach: Evidence of Unclear and Present Danger
Source: PLoS One. 2014 Oct 15;9(10):e109988. doi: 10.1371/journal.pone.0109988 (PMC4198184; doi:10.1371/journal.pone.0109988)
Supplement: Appendix S1 — Assumptions & Constraints. (DOCX) [file pone.0109988.s005.docx]

**Appendix S1. ASSUMPTIONS & CONSTRAINTS**

Real-time evaluation of the behavior of free-ranging deer to our experimental approaches was possibly affected by the likelihood of prior exposure to vehicles, repeated exposure of individuals to the same experimental protocol (i.e., resulting in possible dependence between observations within and between treatments), and our limited control for location and sample size relative to approach speed. We contend, however, that our experimental protocol reduced the likelihood of double sampling on the same night. Our adherence to at most one night of experimental approaches per week further reduced any effect of multiple observations on the same individuals within and between treatments. Runyan & Blumstein [1] suggested that researchers need not be concerned about individual animal identity when studying variables generally dependent upon environmental factors, as opposed to properties of individuals (e.g., habituation) relative to treatment [but see 2]. In addition, we assumed that the probability of observing behavior of experimentally naive individuals would be equivalent across approach speeds and over time. We also considered that the potential effect of dependence of observations relative to approach speeds, as well as the potential effect of the periodic exposure of naive individuals, would be constant over time. Relative to sample size, although we randomized with regard to route, direction, and maximum approach speed, we could not maintain a balanced set of observations between approach speeds simply because of the random aspect of where deer might be encountered and whether we could accelerate to the pre-selected maximum approach speed on a particular section of a route.

As noted earlier, an obvious constraint imposed by the logistics of the experiment was our inability to detect and record alert response to vehicle approach. More specifically, alert response relative to distance of the approaching vehicle would have provided us means to better quantify the effect of start distance and the potential confounding issue of noise associated with acceleration. Another constraint was the issue of detecting deer adequately at night so as to allow enough time to reach highway speeds (i.e., speeds generally > 89 km/h). An enhanced ability to detect deer might well have yielded greater sample sizes within smaller intervals of speed and, thus, a finer-scale examination of the effects of start distance and speed. Further, our experiment was conducted on roads ≤6.1 m in width and experiencing negligible traffic volume at night. Road configuration, including width, access, and egress to cover, as well as traffic volume and speed are critical factors, among others, contributing to animal-vehicle collisions [3], [4].

Also, Carrete & Tella [2] report that individual consistency in FID relative to variation in individual susceptibility to disturbance can influence interpretation of FID within the experimental context, and subsequently affect management options (e.g., buffer distances in conservation areas). Specifically, individual temperament might affect how animals distribute themselves relative to levels of disturbance [5], such that more disturbance-tolerant individuals might reside closer to areas of human disturbance [2]. Further, animals might also adjust their FID relative to learned levels of vehicular traffic and speed (e.g., European passerines) [6]. Distribution of deer populations by temperament would not eliminate DVCs, but provides a possible explanation for our findings versus differential deer response to varying levels of threat posed by actual predators or an approaching human (e.g., mule deer response to human approach and behavior) [7]), or other disturbances [8]. Also, adaptation to levels of vehicle traffic and speed on PBS, *sensu* [6] might reduce variability in FID. We note, however, that our analysis focused on quantiles of the raw data, not central tendencies of the response variables (i.e., FID and TTC), and thus likely captured individual variation in perception and response to risk posed by vehicle approach.

**References**

1. Runyan AM, Blumstein DT (2004) Do individual differences influence flight initiation distance? J Wild Manage 68: 1124–1129.
2. Carrete M, Tella JL (2010) Individual consistency in flight initiation distances in burrowing owls: a new hypothesis on disturbance-induced selection. Biol Lett 6: 167–170.
3. Forman RTT, Sperling D, Bissonette JA, Clevenger AP, Cutshall CD, et al., editors (2003) Road ecology. Science and solutions. Washington DC, USA: Island Press.
4. Fahrig L, Rytwinski T (2009) Effects of roads on animal abundance: an empirical review and synthesis. Ecol Soc 14: 21.
5. Martin JGA, Réale D (2008) Animal temperament and human disturbance: implications for the response of wildlife to tourism. Behav Processes 77: 66–72.
6. Legagneux P, Dcatez S (2013) European birds adjust their flight initiation distance to road speed limits. Biol Lett 9: 20130417.
7. Stankowich T, Coss RG (2005) Effects of predator behavior and proximity on risk assessment by Columbian black-tailed deer. Behav Ecol 17: 246–254.
8. Stankowich T (2008) Ungulate flight responses to human disturbance: a review and meta-analysis. Biol Cons 141: 2159–2173.
