# Supplementary material for: White-Tailed Deer Response to Vehicle Approach: Evidence of Unclear and Present Danger
Source: PLoS One. 2014 Oct 15;9(10):e109988. doi: 10.1371/journal.pone.0109988 (PMC4198184; doi:10.1371/journal.pone.0109988)
Supplement: Appendix S2 — Deer Response to Vehicle Approach & Lighting. (DOCX) [file pone.0109988.s006.docx]

**Appendix S2. DEER RESPONSE TO VEHICLE APPROACH AND LIGHTING**

Based on personal observations of the authors, deer habitat use on PBS and behavior relative to vehicle traffic during daylight hours differs from that seen at night. During daylight hours, deer generally more concealed and located farther away from roads. As few personnel are present on PBS at night and vehicle traffic is decreased, deer tend to use roadway margins.

As for response to vehicle approach at night, Blackwell and Seamans [1] reasoned that a vehicle-based lighting system that better complemented peak visual capabilities of white-tailed deer at night relative to standard tungsten-halogen (TH) lighting alone would elicit a greater flight-initiation distance (FID) by free-ranging deer. The following material represents modified text from Blackwell and Seamans [1].

The authors used 2 TH lamps and one Xenarc high-intensity discharge (HID) lamp. The HID lamp better approximated full-spectrum light (Maximum spectral irradiance, MSI, at 476.41 nm; Total spectral irradiance, TSI, 400–700 nm = 0.74 x 10^6^ μW∙cm^-2^/nm), including approximately 40.4% (0.30 x 10^6^ μW∙cm^-2^/nm) of TSI from 400 nm to 537 nm. Thus, the HID lamp produced a TSI that was 51% of the TH lamps combined and better complemented the SWS and MWS cones and rod pigment (peak absorption from 400 nm to 537 nm) within the retina of white-tailed deer [2], [3]. The combination of TH lamps with the HID lamp on constant illumination yielded an MSI at 546.46 nm and an increase in TSI by 27.5% (TSI ¼ 1.83 3 106 lW/cm2/nm). In addition, approximately 33.8% (0.62 x 10^6^ μW∙cm^-2^/nm) of the combined TSI was from 400 nm to 537 nm. Also, the combined system increased the absolute amount of spectral irradiance (400–537 nm) by 34.8%. The 2-Hz pulse of the HID lamp modulated the spectrum between the TH lamps and the combined spectra.

Blackwell and Seamans [1] modeled deer FID relative to vehicle starting distance, lighting treatment, season, and deer group size. Vehicle approach speed was a constant 40.2 km/hour. Deer exposed to the combination of TH lamps and constant illumination of the HID lamp exhibited a mean (SD) FID of 136 (127) m. In contrast, deer exposed to TH lamps only initiated flight on average at 116 (127) m, and those exposed to the combination of TH lamps and the HID lamp pulsed at 2 Hz exhibited a mean FID of 89 (98) m. The authors contended that the pulsing of the HID lamp while TH lamps were illuminated resulted in consistent loss (over approx. 0.5-sec intervals) of a portion of the image on approach, possibly interfering with sensory information relative to the position of the potential threat. In contrast, the combination of TH lamps and constant illumination of the HID lamp contributed significantly to the probability of a FID ≥94 m.

**References**

1. Blackwell BF, Seamans TW (2009) Enhancing the perceived threat of vehicle approach to white-tailed deer. J Wildl Manage 73: 128–135.
2. Neitz J., Jacobs GH (1989) Spectral sensitivity of cones in an ungulate. Vis Neurosci 2: 97–100.
3. Jacobs GH, Deegan JF II, Neitz J, Murphy BP, Miller KV, et al. (1994) Electrophysiological measurements of spectral mechanisms in the retinas of two cervids: white-tailed deer (Odocoileus virginianus) and fallow deer (Dama dama). J Compar Phys A 174: 551–557.
